# A Therapeutic Strategy for Lower Motor Neuron Disease and Injury Integrating Neural Stem Cell Transplantation and Functional Electrical Stimulation in a Rat Model

**DOI:** 10.3390/ijms23158760

**Published:** 2022-08-06

**Authors:** Katsuhiro Tokutake, Masaru Takeuchi, Shigeru Kurimoto, Sota Saeki, Yuta Asami, Keiko Onaka, Masaomi Saeki, Tadayoshi Aoyama, Yasuhisa Hasegawa, Hitoshi Hirata

**Affiliations:** 1 Department of Human Enhancement and Hand Surgery, Nagoya University Graduate School of Medicine, 65 Tsurumai-cho, Showa-ku, Nagoya 466-8550, Japan; 2 Department of Micro-Nano Mechanical Science and Engineering, Nagoya University Graduate School of Engineering, Furo-cho, Chikusa-ku, Nagoya 464-8603, Japan

**Keywords:** peripheral nerve injury, Wallerian degeneration, spinal motor neuron, cell transplantation, denervated muscle, functional electrical stimulation (FES), Schwann cells, regeneration, muscle reinnervation, neural interface

## Abstract

Promising treatments for upper motor neuron disease are emerging in which motor function is restored by brain–computer interfaces and functional electrical stimulation. At present, such technologies and procedures are not applicable to lower motor neuron disease. We propose a novel therapeutic strategy for lower motor neuron disease and injury integrating neural stem cell transplantation with our new functional electrical stimulation control system. In a rat sciatic nerve transection model, we transplanted embryonic spinal neural stem cells into the distal stump of the peripheral nerve to reinnervate denervated muscle, and subsequently demonstrated that highly responsive limb movement similar to that of a healthy limb could be attained with a wirelessly powered two-channel neurostimulator that we developed. This unique technology, which can reinnervate and precisely move previously denervated muscles that were unresponsive to electrical stimulation, contributes to improving the condition of patients suffering from intractable diseases of paralysis and traumatic injury.

## 1. Introduction

Loss of lower motor neurons (LMNs) due to spinal cord injury, extensive brachial plexus injury, or neurodegenerative disease causes irreversible skeletal muscle atrophy and paralysis. People who suffer from such a debilitating condition experience resultant physical limitations and psychological burdens as well as immeasurable social and economic losses. In cases of severe traumatic nerve injury, various surgical treatments such as nerve repair, nerve grafting, tendon transfer, nerve transfer, and free functional muscle transfer have been used to in an attempt to reconstruct a functional motor system, but the results have never been fully satisfactory [1]. Furthermore, progressive motor neuron diseases such as amyotrophic lateral sclerosis (ALS), which affect tissues involved in fundamental processes such as breathing and swallowing, remain intractable [2].

In recent years, stem cell transplantation, including of induced pluripotent stem (iPS) cells, has been the focus of much research, with the goal being to administer these cells to central nervous system (CNS) tissues to replace or relay neurons or to promote neurotrophic factors and remyelination [3]. High-level spinal cord injury, a leading cause of refractory paralysis, often results in disruption of upper extremity function. In spinal cord injury, both upper motor neuron (UMN) and LMN involvement is common [4]. Patients with irreversible conus and cauda equina lesions caused by spinal cord injury also have severe paralysis of leg muscles due to LMN denervation [5]. When LMNs are involved, functional recovery is time-constrained because regenerating axons must reach the target muscle in 12 to 18 months. However, it is difficult for transplanted CNS neurons to extend axons from the spinal cord to the target muscle, which is sometimes 20,000 times longer than the cell body, in order to reinnervate the denervated muscle and replace LMNs. If this does not occur and CNS neurons are unable to regain control, disruption of neuromuscular junctions and irreversible muscle atrophy is the result. Neuron replacement has not been shown to be an effective treatment for LMN disease. In ALS, for example, the role of stem cell transplantation is limited to supporting maintenance of the microenvironment [6].

Epidural electrical stimulation devices have been used successfully to target residual LMNs to assist in controlling paraplegia caused by spinal cord injury [7]. Innovative advances in neuroengineering are providing clues to new therapeutic approaches. However, therapeutic strategies that use electrical stimulation are ineffective when neurons are damaged or degenerated. Extensive damage to LMNs and neurodegenerative diseases remain formidable unresolved issues. For example, a clinical intervention for ALS patients using invasive diaphragm pacing to ameliorate potentially fatal respiratory complications was abandoned because, instead of reducing complications, it shortened survival time compared to noninvasive management [8].

During LMN denervation, the neuromuscular junction and muscle fibers gradually degenerate. The degenerating muscle fibers eventually become fibro-fatty, resulting in reduced responsiveness to electrical stimulation and the inability to achieve muscle contraction. The European research group reported that early introduction of home-based FES can prevent severe muscle atrophy and contribute to pressure ulcer prevention, even in permanent denervated muscles. However, very intense stimulation is required for denervated muscles, limiting its applicability [5]. New therapies could emerge synergistically between successful reconstruction of denervated muscles and expanding the applications for functional electrical stimulation (FES).

In 1993, Erb et al. first described transplanting embryonic spinal motor neurons into peripheral nerve trunks to forestall the otherwise irreversible process leading to muscle denervation [9]. They showed that transplanted cells can be viable within the peripheral nerve and that axons extending from the neuronal cell body can form neuromuscular junctions within the denervated muscle. Several follow-up studies have been published since then, which reveal that this approach prevents denervated muscle atrophy and creates motor units in peripheral tissues that contract with electrical stimulation, even though there is no continuity with the central nervous system [10,11,12].

We are the first to report that the combination of neural stem cell transplantation into peripheral nerves and FES can improve motor function [13]. However, the FES strategy for drop foot reported in the paper had limitations, namely: the risk of infection by wires, the difficulty of selective stimulation of nerves, the need for manual manipulation, the impossibility of complex movements, and the inability to modulate stimulation incrementally. To overcome these challenges, our researchers developed a wirelessly powered two-channel neurostimulator that modulates switching according to the frequency of the transmitted signal and the controlled angle of the naive ankle joint by changing the stimulation electrode and current using a visual feedback control system based on the proportional–integral (PI) control method [14].

**Figure 1:**
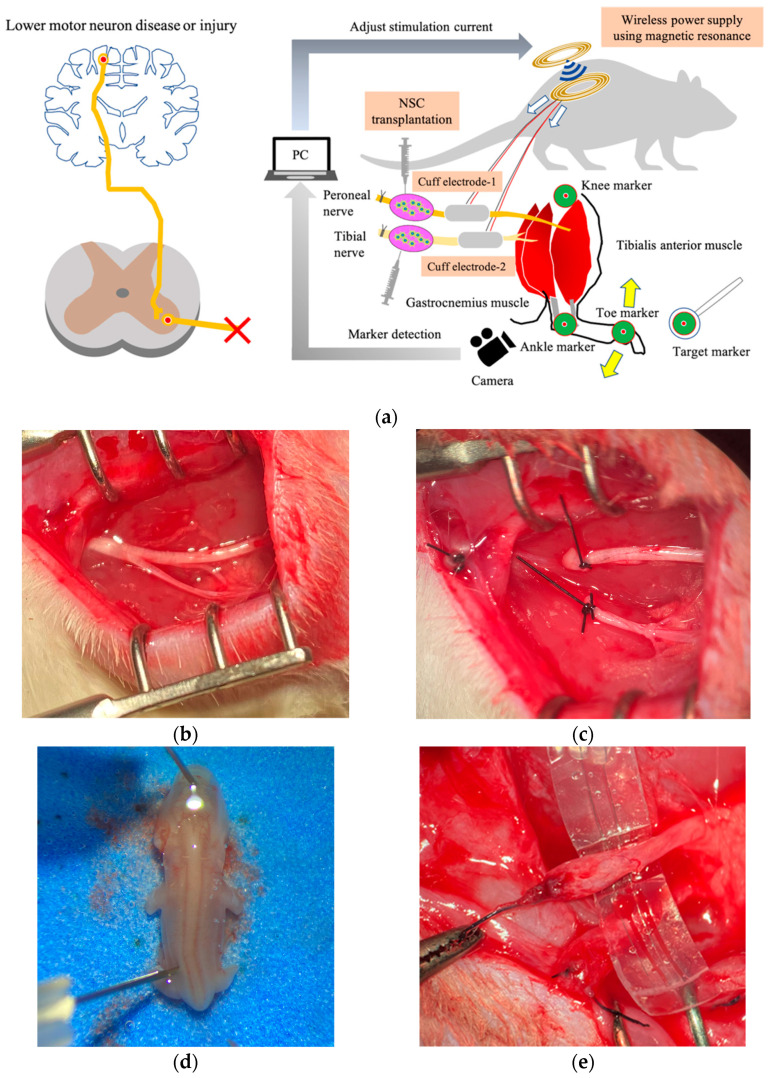
Novel therapeutic strategy for denervated muscles. (**a**) Scheme of denervated muscle reinnervation by stem cell transplantation and visual feedback control. (**b**) Exposure of the tibial and common peroneal nerves branching from the sciatic nerve in the thigh. (**c**) Ligation and transection of the sciatic nerve and the tibial and common peroneal nerves. (**d**) Collection of embryonic spinal cord-derived neural stem cells on E14. (**e**) Cuff electrode as interface and distal stump of nerve with ectopic ganglion four months after transplantation.

However, it is unclear whether computer control using muscle synergy of multiple denervated muscles that have been reinnervated by stem cell transplantation into peripheral nerves can achieve smooth movement and precise angle control of multiple joints. In this study, we present a therapeutic strategy to move and control denervated muscles integrating regenerative medicine, FES, a visual feedback system, and a wireless power supply in a rat sciatic nerve transection model (Figure 1).

## 2. Results

### 2.1. Neural Stem Cells Transplanted into Peripheral Nerves in Denervated Muscle Formed a Complete Peripheral Motor Unit That Contracted upon Electrical Stimulation

#### 2.1.1. Tissue Analysis

**Figure 2:**
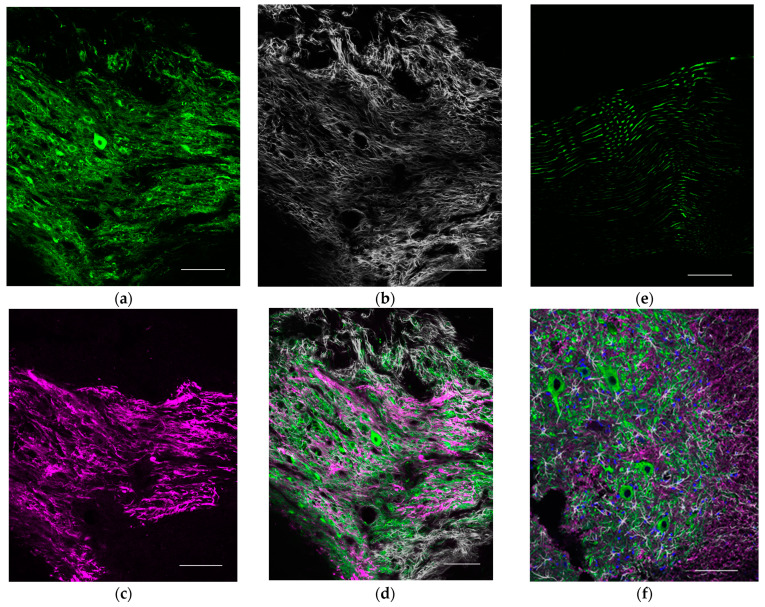
Immuno-histological evaluation at the transected distal peripheral nerve with transplanted cells compared to the naive nerve and spine (sagittal section). (**a**) Tuj1 (β3 tubulin) staining. (**b**) GFAP (glial fibrillary acidic protein) staining. (**c**) MBP (myelin basic protein) staining. (**d**) Tuj1/GFAP/MBP staining. (**e**) Tuj1 staining in the intact peripheral nerve without transplantation. (**f**) Tuj1/GFAP/MBP staining at the anterior horn of the spinal cord. Scale bar = 100 μm.

Immunostaining of the transected distal peripheral nerve with transplanted cells revealed central nervous system cells such as neurons (Tuj1; β3tubulin positive), astrocytes (GFAP positive), and MBP-positive cells thought to be oligodendrocytes or Schwann cells, forming nodular structures (Figure 2). This indicates that the transplantation formed ectopic ganglia similar to those in the central nervous system.

**Figure 3:**
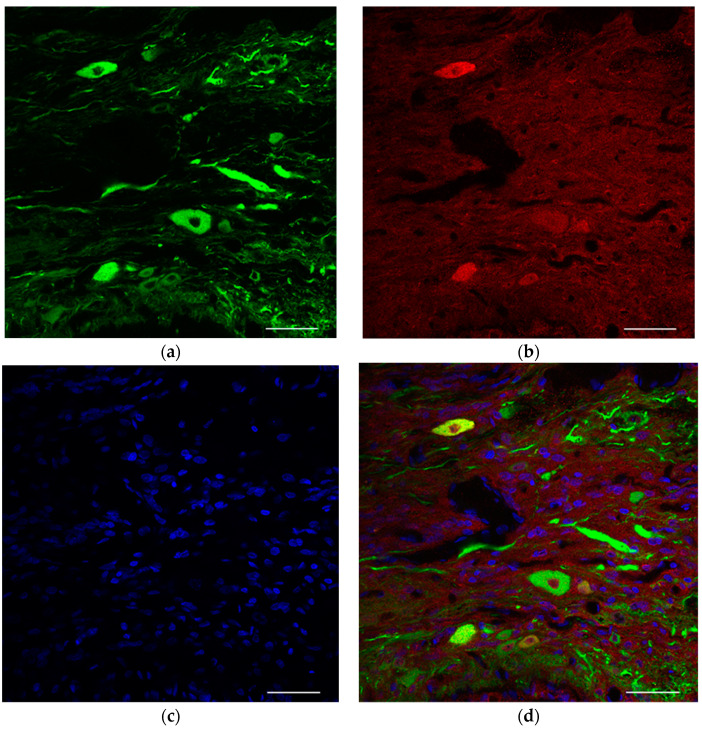
Immunohistological analysis of survival-engrafted neuron at the end of the distal peripheral nerve (sagittal section). (**a**) Tuj1 (β3 tubulin) staining. (**b**) ChAT (choline acetyltransferase) staining. (**c**) Hoechst staining. (**d**) Tuj1/ChAT/Hoechst staining. Scale bar = 50 μm.

ChAT/Tuj1-positive cells were also identified among the neurons (Figure 3). Considering their ventral spinal cord origin, these cholinergic neurons were possibly motor neurons. Within normal peripheral nerves, no soma was present and only axons were stained with Tuj1, indicating that cell transplantation has resulted in motor neuron engraftment within the distal stump of peripheral nerves.

**Figure 4:**
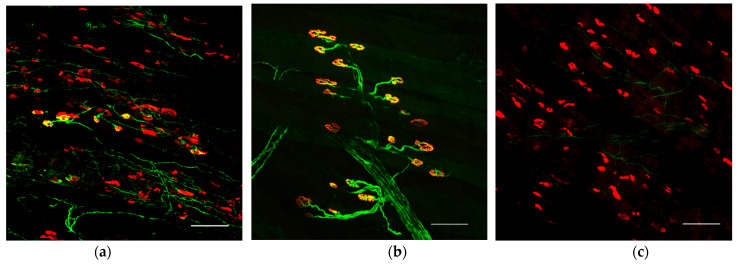
Immunohistological evaluation of the neuromuscular junction of extensor hallucis longus (EHL) in (**a**) transplanted limbs (reinnervated axons enter acetylcholine receptors and form neuromuscular junctions), (**b**) normal limbs, and (**c**) the surgical control limb (without transplantation). Green: Tuj1 (βIII tubulin), Red: αBTX (α-bungarotoxin). Scale bar = 100 μm.

Neuromuscular junctions in the reinnervated extensor digitorum longus muscles were evaluated using fluorescence immunohistochemistry. Regenerative axons labeled with the βIII tubulin neuronal marker reached the neuromuscular junction region where α-bungarotoxin-labeled acetylcholine receptor clusters were located. On the other hand, in the surgical control group, no axons reached the neuromuscular junctions (Figure 4).

**Figure 5:**
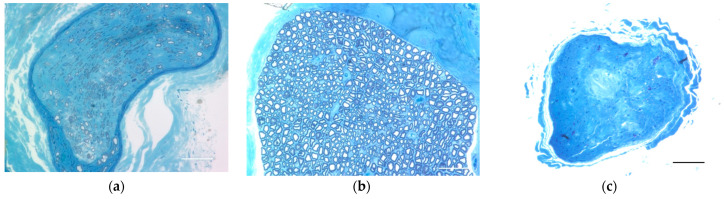
Nerve cross-sections from close to the muscle and distal to the cell transplantation, stained with toluidine blue, show myelinated axons in (**a**) the transplanted nerve and (**b**) the intact nerve, but none in (**c**) the surgical control (without transplantation). Scale bar = 50 μm.

The transplantation group had multiple myelinated axons, although fewer nerves than normal, whereas the common peroneal nerve in the surgical control group had no myelinated axons (Figure 5).

#### 2.1.2. Electrophysiological Evaluation

**Figure 6:**
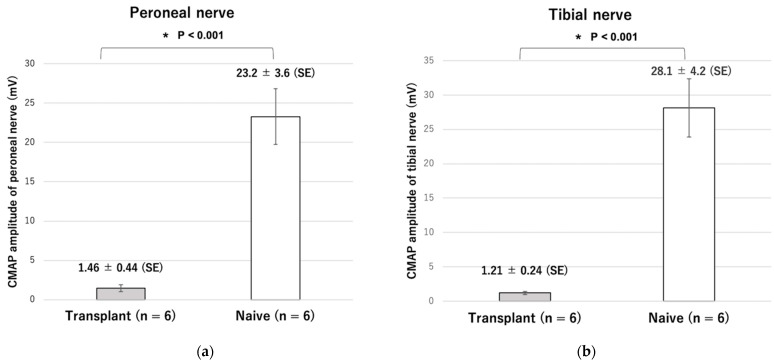
Amplitude of the compound muscle action potential (CMAP) of the (**a**) peroneal nerve and (**b**) tibial nerve. * *p* < 0.05.

Although the amplitude of CMAP was approximately 1/15–1/20 that of the naive group (n = 6), nerve stimulation in the transplantation group (n = 6) resulted in the contraction of all muscles (Figure 6).

**Figure 7:**
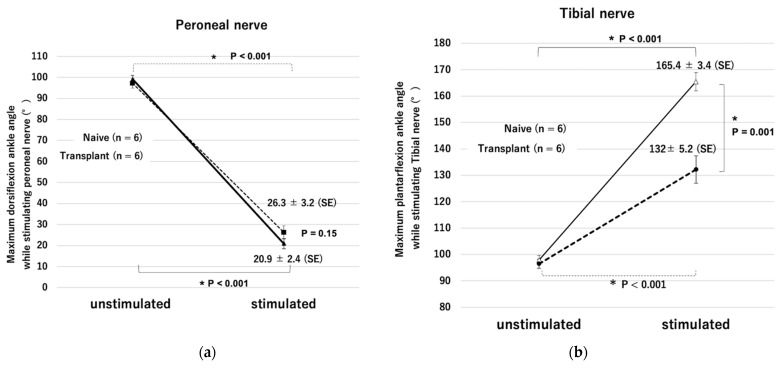
Changes in maximum ankle angle during electrical stimulation. (**a**) Maximum dorsiflexion while stimulating the peroneal nerve. (**b**) Maximum plantar flexion while stimulating the tibial nerve. * *p* < 0.05.

**Figure 8:**
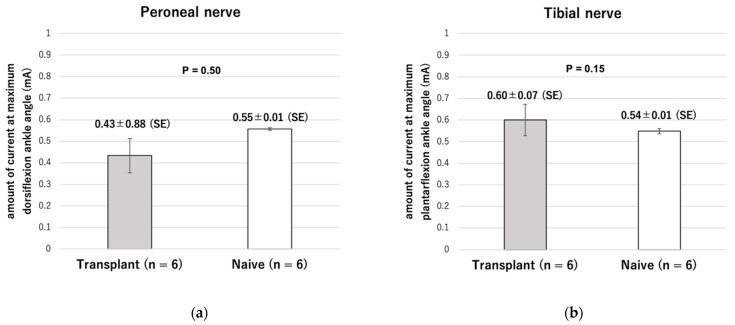
Amount of current applied to attain the maximum ankle angle. (**a**) For maximum dorsiflexion while stimulating the peroneal nerve. (**b**) For maximum plantarflexion while stimulating the tibial nerve.

In the naive group, the dorsiflexion angle changed from 98° to 20° from unstimulated to stimulated state (78° difference). In the transplant group, the angle changed from 95° to 26° (a 69° difference). Both angles of stimulated state changed more than the angle of unstimulated state with statistically significant differences (*p* < 0.001) and the amount of current required was not significantly different (0.43 ± 0.08 mA in the transplantation group, 0.55 ± 0.006 mA in the naive group, *p* = 0.15). Plantar flexion movements were also observed in all six rats, but the change in range of motion was significantly smaller than that in the naive group. There was no difference in the amount of current required (0.60 ± 0.07 mA in the transplantation group and 0.54 ± 0.01 mA in the naive group, *p* = 0.50) (Figure 7 and Figure 8). The maximum ankle angle was obtained, even in the transplantation group, using low-power stimulation equivalent to that in the naive group.

**Figure 9:**
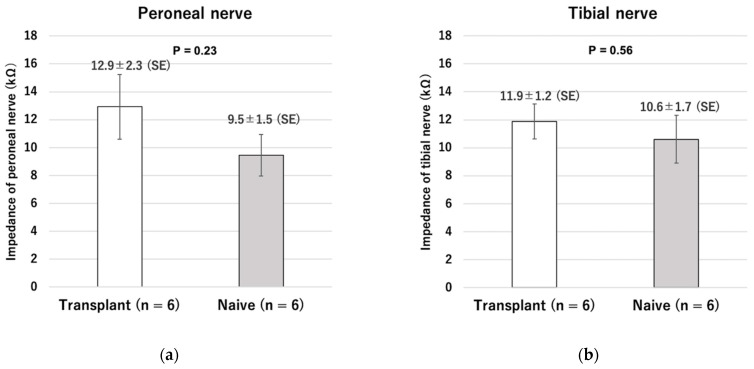
Comparison of nerve impedance between transplanted nerve and intact nerve. (**a**) Peroneal nerve. (**b**) Tibial nerve.

There was no significant difference in the impedance of the stimulated nerves between the transplantation and naive groups (common peroneal nerve: transplantation group 12.9 ± 2.3 kΩ, naive group 9.5 ± 1.5 kΩ, *p* = 0.23; tibial nerve: transplantation group 11.9 ± 1.2 kΩ, naive group 10.6 ± 1.7 kΩ, *p* = 0.56) (Figure 9).

### 2.2. Visual Feedback Control of Previously Denervated Muscles Can Achieve Smooth Movement and Precise Angle Control

In a surgical control without transplantation, four months after losing innervation of LMNs from the spinal cord, the lower limb did not respond or move at all with electrical stimulation. However, in the six cell transplanted limbs, the motor unit was reconstituted and responded to electrical stimulation.

**Figure 10:**
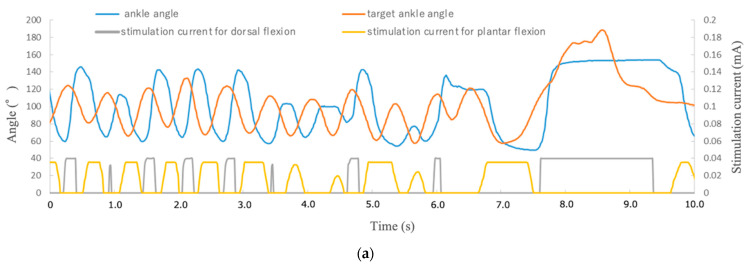

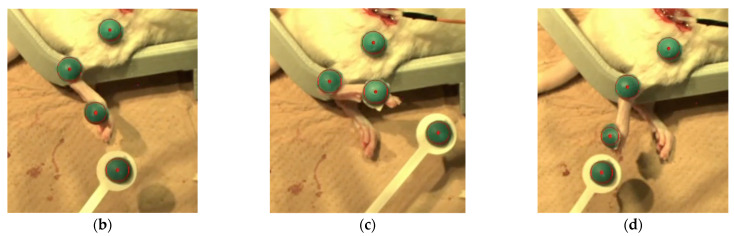
Visual feedback control for reinnervated muscles (previously denervated muscles). (**a**) Relationship between ankle joint tracking with the target and stimulation current. (**b**) No stimulation. (**c**) During dorsiflexion. (**d**) During plantar flexion.

Visual feedback control of rat limbs for 10 random movements with target markers showed that even denervated muscles reinnervated via ectopic ganglia could follow and control target markers quickly and precisely by applying the appropriate amount of current to the distal stump of the nerve (0.25 s average time difference between movement of target marker and limb; 7.96° difference in angle at the target point) (Table 1, Figure 10).

Video S1 as Supplementary Materials shows reinnervated muscles following a randomly moving target by visual feedback control (including the surgical control groups).

## 3. Discussion

In the present study, we were able to visualize motor neurons surviving in peripheral nerves as a result of stem cell transplantation. This enabled reinnervation of previously denervated muscles. We speculate that elongating axons formed new neuromuscular junctions that enabled the denervated muscle to respond to electrical stimulation.

In previous studies, neurostimulators used in healthy rat limbs showed step responses of 200–250 ms [14,15]. The denervated muscle reinnervated by stem cell transplantation in the present study had a similar response, and there was little oscillation generation and good precision of control in the visual feedback control tracking of the randomly moving target, as shown in the Supplementary Video S1. After cell transplantation, the distal nerve stump with ectopic ganglia also showed no statistically significant difference in impedance compared to normal nerves. Moreover, even in the transplantation group, the maximum ankle angle could be obtained by using the low-power stimulation equivalent to that required to attain the maximum angle in the naive group. These results indicate that the ectopic ganglia of the peripheral distal nerve stump and reinnervated muscle can be connected and adapted to our previously reported visual sensory feedback system using cuff-type electrodes as an interface.

Liberson et al. achieved functional recovery of drop foot due to paralysis using surface electrodes in 1961 [16]. Recent advances in bioengineering have led to the rapid development of medical technologies, including in FES systems, which are breakthroughs in treating paralysis. For example, Ajiboye et al. developed a FES system with a brain–computer interface (BCI) to assist an affected person to regain reaching and grasping limb movements [17]. In their proof-of-concept experiment, FES was used by a tetraplegic participant with a high-level spinal cord injury to spontaneously control his paralyzed upper limb to extend his arm, grasp a cup with his hand, raise it to his mouth, and drink from it. It showed that motor commands could be extracted from the brain and used to control a paralyzed limb by electrical signals.

Bouton et al. refer to this process of extraction and stimulation using BCI and FES techniques as an “electronic neural bypass”, which is based on the idea that electrical signals can replace the role of the neuronal relay. They reason that it will develop further given technological advancements [18]. In fact, the wearable garment-based stimulation technology and electroencephalogram (EEG)-based BCI have been implemented clinically [19]. However, the therapeutic strategy is only indicated if LMNs are still responsive to electrical stimulation.

For patients with complete conus and cauda equina lesions which can cause permanent denervation of muscles, the European research group has demonstrated that it is possible to contract denervated muscles with longer pulse widths and stronger stimulation from surface electrodes than usual. These studies have produced very important evidence on denervated muscles, such as the time course of muscle atrophy in completely denervated human muscles, the establishment of CT scans as a diagnostic method, and the improvement of skin thickness by FES [5,20,21,22,23]. However, a challenge with regard to clinical application is the need for continuous and high-intensity electrical stimulation of the denervated muscle, which requires power supply control that produces a higher delivery energy than nerve stimulation.

The most remarkable achievement of the present study is that the application for FES has been expanded to include reinnervating denervated muscles, which otherwise do not respond to normally electrical stimulation. By reinnervating the denervated muscle, muscle contraction can be obtained with a relatively small current supply. The integration of regenerative medicine using spinal motor neuron transplantation and advanced FES will enable new forms of motor reconstruction offering people affected by LMN disease or injury who lack continuity with the central nervous system the ability to intentionally regain control over their previously denervated muscles.

In this study, central nervous system cells, including glial cells, formed ganglia in the transplanted peripheral nerves even though they were disconnected from the central nervous system. Fundamentally, humans perform highly complex motions through largely subconscious processes, without directing or awareness of specific joint movements or muscle contractions. It is impossible to instantaneously determine the coordinated contraction and tension of multiple primary and antagonist muscles and then consciously orchestrate and direct individual muscles to act. Many limb motions are performed unconsciously in a coordinated pattern called muscle synergy [24] and in specific movement patterns called central pattern generator (CPG) networks [25]. In fact, in insects such as Drosophila, a distributed control system using ganglia in each segment is common, with the central nervous system integrating external information and functioning as a transmitter using command-type neurons [26]. When the feedback control described in the present study is combined with feedforward control based on many specific movement patterns and a system sensing the impetus to move, this strategy will reach its potential.

A recent review article focused on neuromuscular preservation for denervated muscles using spinal motor neuron (SMN) transplantation and discussed permanent treatment strategies with electrical stimulation [27]. In the article, the authors discuss not only reconstruction of motor function due to complete conus and cauda equina injury, but also reconstruction of bladder function and application of diaphragmatic pacing for ALS. Denervated muscles in peripheral facial nerve palsy are also potential targets for this treatment [28]. A strategy of SMN transplantation coupled with electrical stimulation has the potential to dramatically improve the prognosis for denervated muscles which are currently considered untreatable.

Nakano et al. have demonstrated that direct neurotization of nerve grafts followed by transplantation of embryonic spinal neural stem cells within the graft one week later can similarly reinnervate denervated muscles [29]. This phenomenon implies that cells activated by peripheral nerve injury, i.e., de-differentiated Schwann cells and peripheral nerves as scaffolds, are essential elements. Compared to the poor regenerative capacity of the central nervous system in mammalian adults, the peripheral nervous system has a remarkable ability to regenerate after injury [30].

When peripheral nerves are severed or suffer severe crush injuries, nerve fibers distal to the site of injury undergo Wallerian degeneration. De-differentiated Schwann cells (SCs) proliferate and elongate to line the endoneurial tube. This induces and supports regenerating axons that emerge from the stump of the surviving nerve attached to the soma of the neuron. SCs downregulate myelin-related genes and upregulate growth-related genes, including neurotrophic factors as well as damaged neurons. This sequence of processes may be involved in the survival of neurons and axonal regeneration. However, the gene expression is transient and gradually fails to support axonal regeneration within the endothelial tube containing the SC [31].

Sawada et al. investigated whether functional reinnervation of denervated muscle could be achieved by early or delayed cell transplantation relative to the time of denervation. They reported that the neurons survived and regenerated axons up to 24 weeks after transplantation, but their number gradually decreased after peaking at 1 week. This fact also indicates that de-differentiated Schwann cells are the key to this therapeutic strategy [32]. In this study, similar to previous reports, the CMAP amplitude was lower, and the range of plantar flexion motion was significantly smaller in the stem cell-transplanted reinnervated muscle than in the healthy side.

Grumbles et al. reported long-term delivery of FK506 to muscle enhances reinnervated motor unit strength [33]. They also reported that cell transplantation with glial cell line-derived neurotrophic factor (GDNF), hepatocyte growth factor (HGF), and insulin-like growth factor (IGF) significantly increased motoneuron survival, myelinated axon counts, muscle reinnervation, and evoked electromyographic activity compared to cell transplantation alone [11].

Yang et al. reported that electrical stimulation for 1 h of embryonic neurons at 1 Hz during transplantation improved axonal regeneration and the number of functioning innervated muscles [34]. A more detailed analysis of the environment and molecular mechanisms required for the survival and differentiation of cell transplantation may solve the problems regarding more stable methods of innervation and improvement in muscle contraction.

Electrical stimulation therapy via a peripheral nerve interface has been developed with contributors from various fields. Therapies using the technology have been approved by the US Food and Drug Administration (FDA) [35,36,37,38,39]. Recently, shape memory polymers (SMPs) have been designed to assume a specific shape at body temperature, and studies on 30 μm thin neural interfaces using SMP were demonstrated to minimize damage to nerves and maintain their condition [40]. Technological innovations in materials will lead to the emergence of interfaces that are less invasive while administering stable stimulation. Progress can be seen in wireless and battery-free technologies while developments in closed-loop control by bidirectional communication are among recent advances in neuroengineering [41,42]. Peripheral nerves are present at various locations in the body, and the direct neurotization method using peripheral nerve fragments reported by Nakano et al. can be used to construct ganglia in denervated muscle targeted for reinnervation, while the timing of Schwann cell de-differentiation can also be controlled [29].

This study had several limitations. First, it showed the stimulation status at the time of cuff electrode placement but did not show changes associated with long-term implantation. Second, ankle motion was controlled under anesthetic and non-gravitational conditions and did not involve changes in the surrounding environment except for the target marker. Therefore, we were able to reproduce movements similar to those of healthy limbs, but further improvements will be necessary when more force is required. However, this may present new challenges. For example, as reported in the Project of the European research group, stimulation of continuous denervated muscle contraction has the potential to cause muscle hypertrophy. Third, changes due to fatigue in the reinnervated muscles during continuous stimulation were not evaluated. Fourth, when considering clinical applications, an alternative to embryonic spinal motor neuron should be considered as the cell source for transplantation. In this regard, human iPSC-derived SMNs and transplantation of SMNs using xenografts that have already been clinically applied, such as minipigs, may be considered as a possible method [29].

In conclusion, although many issues remain to be addressed, this unique technology, which can reinnervate and precisely move denervated muscles that are normally unresponsive to electrical stimulation, contributes toward the goal of improving the condition of patients suffering from intractable diseases of paralysis and traumatic injury. It indicates a promising direction for future investigation connected to multifaceted emerging medical engineering technologies.

## 4. Materials and Methods

### 4.1. Animals

All animal experiments and procedures used in this study were approved by the Animal Ethics Research Committee at Nagoya University (registration number M210152-004).

We used nine adult (8-week-old) male Fischer 344 rats (Japan SLC, Shizuoka, Japan). We transected the sciatic nerve at the femoral level on one side and a medium containing dissociated embryonic spinal neurons was injected into the distal stump of the tibial and peroneal nerves one week later (transplant limbs, n = 6). The other side was evaluated without performing any surgery (naive control limbs, n = 6). Three rats had only nerve transection on one side and no transplantation (surgical control, n = 3). Embryonic ventral spinal cord cells were harvested from the 14-day-old embryos of two pregnant Fischer 344 rats.

### 4.2. Model Preparation

All surgical procedures were performed under a surgical microscope with isoflurane anesthesia (2%, delivered by a calibrated vaporizer through a facial mask). In the transplant limbs, the sciatic nerve was completely transected at the midthigh level, and the tibial and peroneal nerves were divided. Then, each stump was ligated using 5-0 nylon and the sciatic nerve stump was sutured into the gluteus muscle to prevent natural regeneration. The naive control limbs did not undergo surgery.

### 4.3. Cell Preparation and Transplantation

One week after nerve transection, pregnant rats were anesthetized, and their embryos were removed from the uterus. The ventral spinal cords were resected under a surgical microscope and cut into small pieces in Hanks’ balanced salt solution (Life Technologies Japan, Tokyo, Japan). Embryonic ventral spinal cord cells were dissociated using dispersed solutions for neuronal cells (Neuron Dissociation Solutions S 297-78101, Fujifilm Wako Pure Chemical Corporation, Osaka, Japan) and suspended in a culture medium (Neuron Culture Medium 148-09671, Fujifilm Wako Pure Chemical Corporation). Our group has previously demonstrated that the cell population at this harvest time includes neural stem cells that have the potential to differentiate into either neural or glial lineages [29]. Recipient rats were anesthetized. One million embryonic ventral spinal cord cells in a 10 μL culture medium were slowly injected into the distal stumps on the transplantation side using a Hamilton syringe with a 30-gauge needle. Nerve swelling, which was evidence that medium was injected into the nerve, was confirmed during administration.

### 4.4. Electrophysiological Evaluation

Four months after cell transplantation, electrophysiological testing was performed on six rats in transplant group and two rats in surgical control group under anesthesia. The distal stumps of the peroneal and tibial nerves in the transplant limbs and the intact peroneal and tibial nerves in the naive control limbs were surgically exposed. Bipolar stimulating electrodes (OA216-081, Unique Medical Corporation, Osaka, Japan) were grasped and fixed using a fixation table with a small manipulator (SC-2/G-2, Nihon Kohden, Tokyo, Japan), and each nerve was stimulated with square waves at a frequency of 50 Hz and a duration of 200 μs (Neuropack S1, MEB-9404, Nihon Kohden, Tokyo, Japan). An insulator was also placed between the muscle and the electrode to prevent direct stimulation. The stimulus intensity was increased from 0.0 to 2.0 mA in a stepwise fashion. The maximum ankle angle was calculated as the angle subtending a line connecting the knee and ankle joints and a second line connecting the ankle joint and metatarsal head [43]. An increasing dorsiflexion angle was understood to be approaching zero.

After ankle movement was confirmed, the compound muscle action potentials (CMAP) of the tibialis anterior and the lateral gastrocnemius muscles were measured at room temperature (24 °C) using a standard nerve evoked potential recording system (Neuropack S1).

### 4.5. Neurostimulator

In this study, a wirelessly powered two-channel neurostimulator developed in our previous study was used to selectively stimulate two distal stumps of nerves in a rat transection model. A magnetic resonance method was employed to conduct wireless powering on the neurostimulator [13]. The selection of nerve stimulation was enabled by alternating the frequency of the transmitter signal providing wireless power. In our device, three different frequencies (90, 100, and 110 kHz) were used to transmit power from the transmitter to receiver using a magnetic resonance method. The receiver stimulated the peroneal nerve to generate dorsal flexion when 90 kHz of wireless power was applied, while it stimulated the tibial nerve to generate plantar flexion at 110 kHz. At 100 kHz, neither nerve was stimulated. Thus, the nerve to be stimulated was selected by adjusting the frequency of the transmitter signal. The stimulation current was controlled by the amplitude of the transmitter signal. The stimulation frequency, duration, and current were all controlled by the proposed method.

The transmitter device was composed of three parts with different functions: (1) a microcontroller to receive information about nerve stimulation and current level from a PC; (2) an oscillator to control the transmitter frequency based on the output of the microcontroller; and (3) a voltage regulator to adjust the stimulation current based on the output from the microcontroller. The transmitter coil used in the transmitter system was the same as the receiver coil (diameter = 18 mm, self-inductance = 26 μF, and quality factor = 25).

### 4.6. Cuff Electrode Interface

Cuff electrodes fabricated by photolithography were used following the method described in our previous study [13]. We used a cuff electrode made of liquid silicone rubber (MED-4801, Avantor, Radnor, PA, USA), which can be used in human implantation and stainless-steel wire of 50 μm diameter. The cuff electrode was the interface connecting nerves and the device. Two cuff electrodes from the two-channel neurostimulator were connected to the distal stumps of the tibial and peroneal nerves after neural stem cell transplantation to control plantar and dorsal flexion, respectively, of the ankle joint. The cuff electrodes were wrapped around the nerves and sutured with 8-0 nylon under a microscope to obtain stable contacts between the nerve surfaces and stainless-steel wires. After suturing, we confirmed that stable stimulation was possible.

### 4.7. Visual Feedback Control, Assessment of Accuracy, and Nerve Impedance

To achieve feedback control of the angle of the ankle joint, the leg position needed to be detected. A camera (C-ST, Photron, Tokyo, Japan) was used to detect the angles of the ankle joints which had been labeled with markers placed on the metatarsal head, ankle, and knee. Another was used as a target marker to indicate the angle in the rat ankle joint by moving it manually. The sampling frequency of the high-speed camera was 250 Hz, and the frequency of application of the stimulation current was controlled at 12 Hz in our visual feedback system. The control frequency was restricted by the serial communication speed from the PC to the microcontroller (Arduino Uno R3; open-source hardware, and Arduino IDE 1.8.19; open-source software) in our experimental setup. The transmitter system used 0.25 A, 9.0 V, 2.25 W with a 2.2 mm coil gap to activate the receiver system through wireless power. Visual feedback control of the rat ankle joint was performed with the system we developed. In present experiment, the target marker was manually manipulated while the stimulation currents to the peroneal and tibial nerves were controlled by the proportional integral control method.

We conducted the visual feedback control experiment in six reinnervated rat limbs, directing their movement randomly by means of target markers. Responses to 10 random movements were recorded. Limb performance was evaluated based both on the time differential between the initial movement of the target marker and the limb, and on a comparison between the angle of the target marker and of the limb. For comparison, visual feedback control was also performed in the surgical control limbs.

After the visual feedback control experiment, the impedance of the intact and transplanted nerves was measured using an impedance analyzer (Impedance Analyzer IM3570, Hioki, Nagano, Japan).

### 4.8. Animal Sacrifice and Tissue Embedding

After electrophysiological evaluation and the visual feedback control experiment, nine rats, including the rat serving as surgical control, were sacrificed for tissue analysis. Under isoflurane anesthesia, the left ventricle of each rat was perfused with 50 mL of 0.9% saline, followed by 200 mL of 4% paraformaldehyde in 0.1 M phosphate buffer (pH 7.4). The common peroneal and tibial nerves were harvested for immunohistochemical and histochemical analyses and were divided into the proximal portion, including the transplanted site and the remaining distal portion. The distal portion was prefixed with 4% PFA/2% glutaraldehyde/PBS and then embedded in Epon. The proximal portion was frozen-embedded for immunohistochemical analysis with isopentane, which was cooled with liquid nitrogen after sucrose fixation.

### 4.9. Evaluation of Regenerated Myelinated Axons

The distal portion of the Epon-embedded common peroneal nerve was cut into a 1 μm-thick cross-section with a glass knife and stained with toluidine blue (Sigma-Aldrich, St. Louis, MO, USA) for light microscopy.

### 4.10. Evaluation of the Neuromuscular Junction

To evaluate the structure of the neuromuscular junction and morphology of the end plates, the extensor hallucis longus (EHL), which is innervated by the common peroneal nerve, was harvested under 2% isoflurane anesthesia before perfusion fixation and crushed on glass slides dripping with 4% PFA. Alexa Fluor 488 conjugated Anti-Tuj1 (801203, 1:400, Biolegend, Sam diego, CA, USA) and Alexa Fluor 594-conjugated anti-α-bungarotoxin (B-13423, 1:400; Molecular Probes, Eugene, OR, USA) antibodies and Hoechst (33342, 1:1000; Dojindo, Kumamoto, Japan) nuclear staining were used for whole-mount immunostaining. For comparison, the neuromuscular junction was evaluated in the naive and surgical control limbs. We observed these tissues using a confocal laser microscope (TiE-A1R, Nikon, Tokyo, Japan).

### 4.11. Morphological Evaluation at the Transplantation Site Forming Nodular Structures and Analysis of Survival-Engrafted Neuron

To assess the transplantation site that forms nodular structures, the fixed proximal nerve portions were cut into 30 μm longitudinal frozen sections. To evaluate CNS cells in the transplant environment and the survival of the engrafted neurons, we used the following stains: Alexa Fluor 488 conjugated anti-Tuj1 antibody (801203, 1:400, Biolegend), Cy3 conjugated anti-GFAP antibody (ab49874, 1:400, Abcam, Cambridge, England), anti-MBP antibody (ab40390, 1:200, Abcam), anti-ChAT antibody (AB144P, 1:50 Sigma-Aldrich), and Hoechst (33342, 1:1000, Dojindo) for nuclear staining. Donkey Anti-Rabbit IgG H & L (Alexa Fluor 647) (ab 150075, 1:200, Abcam) for anti-MBP antibody and Donkey Anti-Goat IgG H & L (Alexa Fluor 594) pre-absorbed (ab 150136, 1:200, Abcam) for anti-ChAT antibody were used as secondary antibodies. We also stained the naive spine and common peroneal nerve with the same antibodies for comparison. We observed these sections using a confocal laser microscopy system (A1Rsi and Ti-E, Nikon, Tokyo, Japan).

### 4.12. Statistics

Student’s *t*-tests and paired *t*-tests were used to compare the outcome measures between groups. All statistical analyses were performed using SPSS Statistics version 22.0 software (IBM, Armonk, NY, USA). Results with *p*-values of less than 0.05 were considered statistically significant. All data are described as means ± standard errors (SEs).

## Figures and Tables

**Table 1:** Accuracy of visual feedback control using the novel nerve stimulator during 10 random movements for reinnervated muscles (previously denervated muscles).

Rat	Time between Movement of Target Marker and Limb (s) *	Angle between Target Marker and Limb at the Target Point (°) *
No.1	0.29 ± 0.03	4.5 ± 1.2
No.2	0.29 ± 0.03	10.8 ± 2.3
No.3	0.22 ± 0.01	10.8 ± 1.7
No.4	0.27 ± 0.02	6.4 ± 2.3
No.5	0.22 ± 0.02	9.7 ± 2.6
No.6	0.24 ± 0.01	5.6 ± 1.7

* Number ± standard error.

## Data Availability

Not applicable.

## Supplementary Materials

The supporting information can be downloaded at: https://www.mdpi.com/article/10.3390/ijms23158760/s1.
